# Delayed Clinical Diagnosis of Alström Syndrome in a Resource-Limited Setting: A Case Report From Rural Pakistan

**DOI:** 10.7759/cureus.100092

**Published:** 2025-12-25

**Authors:** Shahab Shahab, Jawad A Khan

**Affiliations:** 1 Cardiology, Midland Metropolitan University Hospital, Birmingham, GBR; 2 Geriatrics, Midland Metropolitan University Hospital, Birmingham, GBR

**Keywords:** alström syndrome, cardiomyopathy, chronic kidney disease, ciliopathy, cone-rod dystrophy, insulin-resistant diabetes, multisystem disorder, pakistan, rare genetic disorder, sensorineural hearing loss

## Abstract

Alström syndrome is a rare autosomal recessive ciliopathy characterized by progressive multisystem involvement, including retinal degeneration, sensorineural hearing loss, insulin resistance, cardiomyopathy, and chronic kidney disease. Diagnosis is often delayed in regions with limited access to specialized care or genetic testing. We report a case of a 24-year-old male from rural Swat, Khyber Pakhtunkhwa, Pakistan, presenting with advanced multisystem manifestations consistent with Alström syndrome. This case highlights the diagnostic challenges in resource-limited settings and the importance of a multidisciplinary clinical approach. It also underscores the influence of social determinants of health, such as geographic isolation, limited availability of specialist care, and socioeconomic constraints on delayed recognition and progression of cardiometabolic and renal complications.

## Introduction

Alström syndrome (AS) is a rare autosomal recessive ciliopathy caused by pathogenic variants in the ALMS1 gene, leading to progressive multisystem involvement [1]. It is classically characterized by early-onset cone-rod dystrophy resulting in visual impairment, progressive sensorineural hearing loss, obesity, insulin-resistant diabetes mellitus, cardiomyopathy, dyslipidemia, hypogonadism, and chronic kidney disease [1-3]. Fewer than 1500 cases have been reported worldwide, underscoring the rarity of the disorder and the diagnostic challenges it presents even in well-resourced healthcare systems [2,4].

Clinical recognition of AS is particularly difficult in low- and middle-income countries due to age-dependent phenotypic progression, variable expressivity, and limited access to subspecialty services [2,4,5]. In resource-limited settings, such as rural regions of Pakistan, additional barriers, including delayed referrals, fragmented healthcare delivery, lack of longitudinal medical records, and the unavailability of molecular genetic testing, frequently result in delayed or missed diagnoses [4,5]. Consequently, patients often present only after advanced multisystem complications have developed, leading to increased morbidity and poorer outcomes.

This case was evaluated and managed at a tertiary care hospital in Swat, Khyber Pakhtunkhwa, Pakistan, where genetic testing for rare inherited disorders is not routinely available. This study aimed to emphasize the importance of establishing a diagnosis of Alström syndrome using validated clinical criteria in settings with constrained diagnostic resources. By highlighting the real-world challenges of diagnosing and managing AS in a population with a high prevalence of consanguinity, this report aimed to improve clinician awareness and support earlier recognition of rare autosomal recessive disorders in similar resource-limited environments.

## Case presentation

In June 2019, a 24-year-old male from rural northern Pakistan presented to a tertiary care hospital in Swat, Khyber Pakhtunkhwa, with uncontrolled diabetes mellitus and lower urinary tract symptoms. His past history included a lifelong learning disability, registered childhood-onset blindness due to progressive visual impairment, and progressive sensorineural hearing loss diagnosed four years earlier. He had been hypertensive for the past three years. A significant family history included the death of a sibling at a young age from diabetes and renal disease. He lived with his parents, was unemployed, and reported no alcohol or tobacco use.

Clinical findings

On examination, the patient’s vital signs revealed a blood pressure of 150/95 mmHg, heart rate of 102 beats per minute, respiratory rate of 20 breaths per minute, temperature of 37.8°C, and oxygen saturation of 96% on room air. He was short in stature, measuring approximately 160 cm, and obese, with a body mass index of 30 kg/m². Xanthelasma was noted on both eyelids, and the testes were markedly small. Bilateral pitting pedal edema was present. Chest auscultation revealed inspiratory crackles, although no cardiac murmurs were heard. These findings were suggestive of multisystem dysfunction.

Laboratory and diagnostic investigations

The patient’s laboratory results and diagnostic assessments are summarized in Table 1. He exhibited markedly elevated blood glucose and HbA1c levels, leukocytosis, normocytic anemia, dyslipidemia, and biochemical evidence of renal impairment. Hepatic transaminases were raised, indicating hepatic involvement. Hormonal evaluation showed low testosterone with low-normal gonadotropins. Urinalysis suggested a urinary tract infection.

**Table 1:** Laboratory results with reference ranges. The table summarizes the patient's laboratory and diagnostic investigations, including measured values, reference ranges, and clinical interpretations. These findings demonstrate multisystem involvement consistent with Alström syndrome, encompassing metabolic dysfunction, renal impairment, hepatic abnormalities, endocrine disturbances, and cardiomyopathy. HbA1c: hemoglobin A1c; LDL: low-density lipoprotein; HDL: high-density lipoprotein; ALT: alanine aminotransferase; AST: aspartate aminotransferase; ALP: alkaline phosphatase; TSH: thyroid-stimulating hormone; LH: luteinizing hormone; FSH: follicle-stimulating hormone; ECG: electrocardiogram; LV: left ventricle; BNP: brain natriuretic peptide

System/investigation	Patient value	Reference range	Interpretation
Glycemic control			
Fasting blood glucose	290 mg/dL	70-110 mg/dL	High
HbA1c	12%	<6%	Poor control
Blood ketones	1.4 mmol/L	<0.6 mmol/L	Mild ketosis
Lipid profile			
Total cholesterol	290 mg/dL	<200 mg/dL	High
LDL cholesterol	190 mg/dL	<130 mg/dL	High
HDL cholesterol	40 mg/dL	>40 mg/dL	Low-normal
Triglycerides	350 mg/dL	<150 mg/dL	High
Renal function			
Serum creatinine	2.1 mg/dL	0.7-1.3 mg/dL	Elevated
Blood urea	52 mg/dL	15-40 mg/dL	Elevated
Urine dipstick	Positive nitrites and leukocytes	Negative	Suggestive of UTI
Liver function			
ALT	95 U/L	10-55 U/L	Elevated
AST	45 U/L	5-40 U/L	Mildly elevated
ALP	110 U/L	40-129 U/L	Normal
Total bilirubin	0.8 mg/dL	0.2-1.2 mg/dL	Normal
Endocrine profile			
TSH	3.8 µIU/mL	0.4-4.2 µIU/mL	Normal
LH	2.5 IU/L	1.5-9.3 IU/L	Low-normal
FSH	3.0 IU/L	1-12 IU/L	Low-normal
Total testosterone	2.1 ng/mL	2.5-8 ng/mL	Low
Cardiac assessment			
ECG	LVH pattern	Normal	Left ventricular hypertrophy
Echocardiogram	Dilated LV, diastolic dysfunction, raised filling pressures	Normal	Cardiomyopathy
Pro-BNP	1500 pg/mL	<125 pg/mL	Very high

Imaging and cardiac assessment

**Figure 1:**
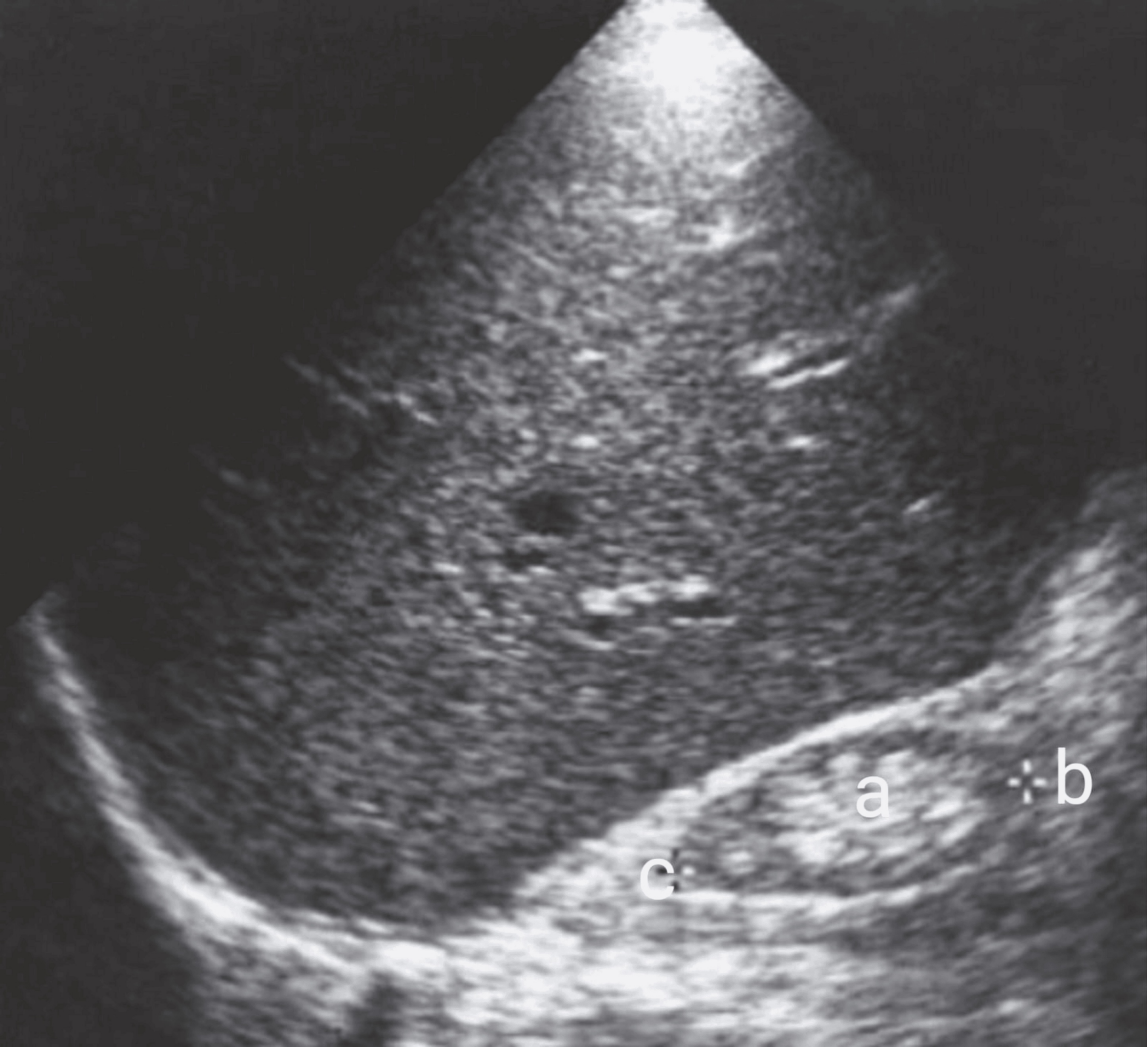
Longitudinal view of the right kidney shows increased cortical echogenicity. (a) A prominent echogenic renal sinus, (b) thinning of the renal parenchyma, and (c) along with overall reduction in renal size, consistent with chronic kidney disease.

**Figure 2:**
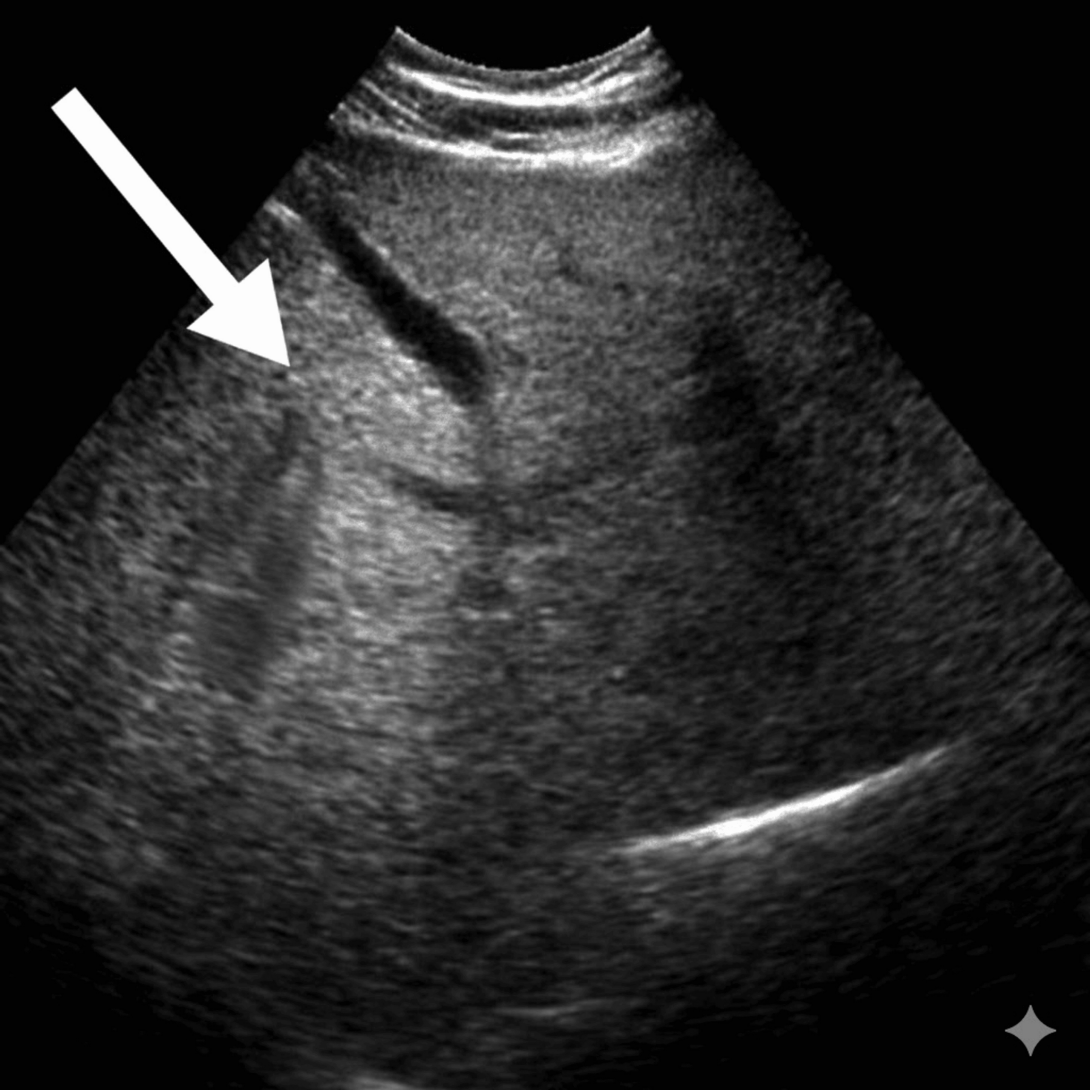
Ultrasound shows increased hepatic echogenicity with poor visualization of the intrahepatic vessel walls (arrow), producing a diffusely bright liver consistent with hepatic steatosis (fatty liver).

**Figure 3:**
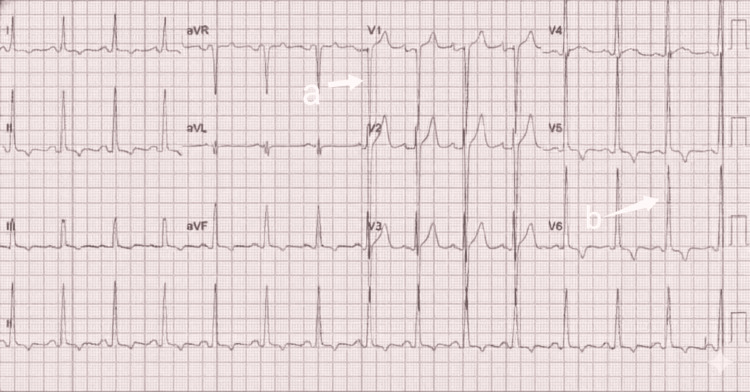
The ECG demonstrates voltage criteria for left ventricular hypertrophy (LVH). In the precordial leads, there is a deep S-wave in lead V1 (arrow a) and a tall R-wave in lead V6 (arrow b).

**Figure 4:**
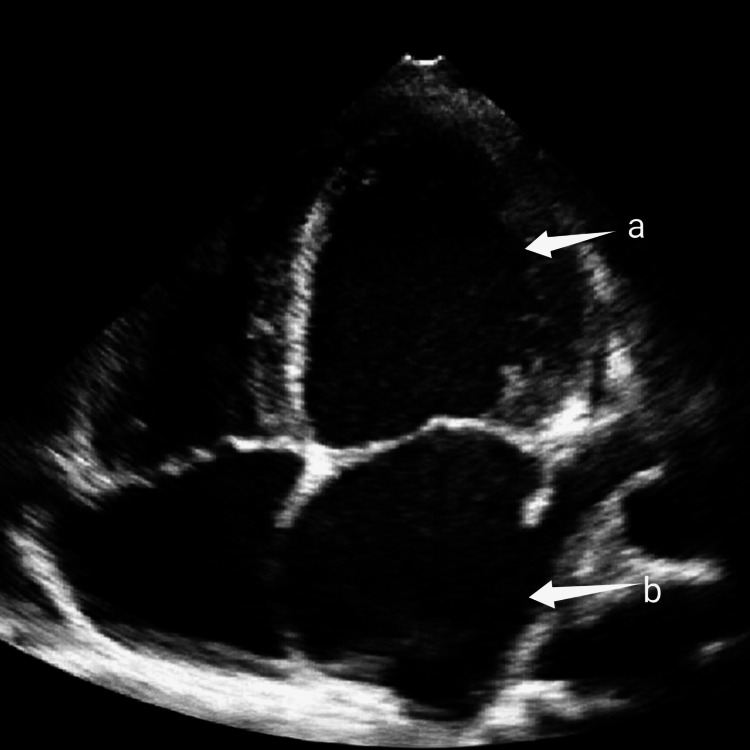
2D echocardiogram apical four-chamber view showing dilated left ventricle (arrow a) and left atrium (arrow b) and eccentric hypertrophy of left ventricle.

Imaging revealed bilaterally small, echogenic kidneys on ultrasound of the kidneys, ureters, and bladder (KUB), consistent with chronic kidney disease (Figure 1). Abdominal ultrasound demonstrated fatty liver without evidence of cirrhosis (Figure 2). Electrocardiography showed a pattern consistent with left ventricular hypertrophy (Figure 3). Echocardiography revealed a dilated left ventricle with significant diastolic dysfunction, elevated filling pressures, and left atrial enlargement (Figure 4).

Diagnosis

Due to the unavailability of genetic testing, the diagnosis was based on the clinical criteria outlined by Marshall et al. [2]. The patient fulfilled the following major criteria: early-onset cone-rod dystrophy leading to blindness, progressive sensorineural hearing loss, and cardiomyopathy with diastolic dysfunction. He also met the following minor criteria: insulin-resistant diabetes mellitus, dyslipidemia, hypogonadism (low testosterone with low-normal gonadotropins), progressive renal dysfunction (chronic kidney disease), and obesity (BMI: 30 kg/m²).

Collectively, the presence of these major and minor criteria strongly supports a clinical diagnosis of Alström syndrome. This explicit listing helps clarify the diagnostic rationale, particularly in resource-limited settings where genetic testing is unavailable.

Differential diagnosis

Bardet-Biedl syndrome (BBS) was considered due to its overlapping characteristics; however, key distinguishing features were absent. The patient had no polydactyly or syndactyly, and the early onset of blindness was more consistent with Alström syndrome, as visual decline in BBS is typically slower. There were no characteristic facial features or evidence of cognitive regression commonly associated with BBS. Additionally, the presence of cardiomyopathy and earlier renal involvement favored Alström syndrome, making BBS less likely.

Management and follow-up

The patient was admitted for management of hyperglycemia and a urinary tract infection. He received intravenous antibiotics, fluid resuscitation, and a variable-rate insulin infusion before transitioning to a regimen of intermediate- and short-acting insulin. Rosuvastatin with ezetimibe was initiated for dyslipidemia, while a beta-blocker and low-dose diuretics were prescribed for cardiac dysfunction. He was referred to endocrinology, nephrology, and cardiology for multidisciplinary follow-up.

At the two-month follow-up visit, fasting blood glucose had improved to 150-170 mg/dL. Renal function remained stable, though impaired. His symptoms of fluid overload had reduced, while visual and hearing deficits remained unchanged.

## Discussion

Alström syndrome (AS) is a rare autosomal recessive ciliopathy characterized by progressive multisystem involvement, considerable phenotypic variability, and age-dependent expression. The constellation of findings observed in this patient, including early-onset visual impairment progressing to blindness, sensorineural hearing loss, insulin-resistant diabetes mellitus, dyslipidemia, cardiomyopathy, hypogonadism, and chronic kidney disease, is consistent with the classical clinical spectrum described in prior cohort studies and case reports [1-3,5].

Visual impairment due to cone-rod dystrophy is typically the earliest manifestation of AS and often presents in infancy or early childhood [1,2]. The early and progressive visual loss in this patient aligns closely with the natural history reported by Marshall et al. and Paisey et al., who identified retinal degeneration as a cardinal feature preceding systemic involvement [2,5]. Similarly, progressive sensorineural hearing loss, commonly developing during childhood or adolescence, was present in this case and mirrors findings reported in multiple longitudinal studies [2,5].

Metabolic abnormalities, including obesity, insulin resistance, type 2 diabetes mellitus, and dyslipidemia, are well-recognized features of AS and generally emerge during adolescence or early adulthood [1,3,6]. In comparison with cases reported from high-income countries, the degree of metabolic dysregulation observed in this patient was notably severe at presentation, with markedly elevated HbA1c and significant dyslipidemia [5,6]. A plausible explanation for this discrepancy is delayed diagnosis and prolonged absence of coordinated metabolic care, which may accelerate disease progression and end-organ damage in resource-limited settings.

Cardiac involvement is a major determinant of morbidity and mortality in AS, with reported phenotypes ranging from infantile-onset dilated cardiomyopathy to adult-onset systolic or diastolic dysfunction [2,7]. The presence of dilated cardiomyopathy with significant diastolic dysfunction in this patient is consistent with adult-onset cardiac involvement described in previous reports [2,7]. However, the advanced cardiac dysfunction at a relatively young age may reflect delayed recognition and lack of routine cardiac surveillance, a contrast to patients diagnosed earlier in well-resourced healthcare systems.

Renal disease in AS is progressive and multifactorial, involving tubular dysfunction, interstitial fibrosis, and eventual chronic kidney disease [3,8]. The finding of small, echogenic kidneys and reduced renal function in this young adult parallels reports of early renal involvement in AS, though the severity appears greater than that described in some series [3,8]. This difference may be attributed to cumulative metabolic insults, recurrent infections, and limited access to nephrology care over time.

Importantly, most published literature on AS originates from high-resource settings where molecular genetic testing enables early confirmation and multidisciplinary management [1,2,5]. In contrast, this case, evaluated at a tertiary care hospital in Swat, Pakistan, demonstrates that reliance on validated clinical diagnostic criteria remains essential where genetic testing is unavailable. The presence of parental consanguinity further strengthens the likelihood of an autosomal recessive etiology and is particularly relevant in regions with high rates of consanguineous marriage, as reported in South Asian populations [4,9].

Overall, while the phenotypic features in this patient are broadly consistent with existing literature, the extent and severity of multisystem involvement at presentation appear more pronounced. This contrast likely reflects systemic barriers in healthcare rather than intrinsic disease variability, underscoring the critical impact of delayed diagnosis, fragmented care, and limited diagnostic resources. This case reinforces the need for heightened clinical suspicion and early multidisciplinary evaluation to mitigate disease progression in resource-limited environments.

## Conclusions

Alström syndrome should be considered in patients presenting with early-onset visual impairment, hearing loss, obesity, diabetes, cardiomyopathy, and renal dysfunction. In settings where genetic testing is unavailable, a diagnosis can be reliably established using validated clinical criteria. Early recognition and a coordinated multidisciplinary approach may help delay organ deterioration and improve patient outcomes. This case underscores the importance of clinician awareness of rare genetic disorders and highlights the challenges of diagnosing and managing Alström syndrome in resource-limited regions such as rural Pakistan.
